# A 96-wells fluidic system for high-throughput screenings under laminar high wall shear stress conditions

**DOI:** 10.1038/s41378-023-00589-x

**Published:** 2023-09-15

**Authors:** Catarina Gonçalves Fonseca, Vânia Silvério, David Barata, Wolfgang Giese, Holger Gerhardt, Susana Cardoso, Claudio Areias Franco

**Affiliations:** 1 Instituto de Medicina Molecular João Lobo Antunes, Faculdade de Medicina, Universidade de Lisboa, Lisboa, Portugal; 2 INESC Microsistemas and Nanotecnologias, INESC-MN, Lisboa, Portugal; 3 Department of Physics, Instituto Superior Técnico, Universidade de Lisboa, Lisboa, Portugal; 4 Max Delbrück Center for Molecular Medicine, Berlin, Germany; 5 DZHK (German Centre for Cardiovascular Research), Partner Site Berlin, Berlin, Germany; 6 Charité – Universitätsmedizin Berlin, Berlin, Germany; 7 Universidade Católica Portuguesa, Católica Medical School, Católica Biomedical Research Centre, Lisbon, Portugal

**Keywords:** Materials science, Nanoscale devices

## Abstract

The ability of endothelial cells to respond to blood flow is fundamental for the correct formation and maintenance of a functional and hierarchically organized vascular network. Defective flow responses, in particular related to high flow conditions, have been associated with atherosclerosis, stroke, arteriovenous malformations, and neurodegenerative diseases. Yet, the molecular mechanisms involved in high flow response are still poorly understood. Here, we described the development and validation of a 96-wells fluidic system, with interchangeable cell culture and fluidics, to perform high-throughput screenings under laminar high-flow conditions. We demonstrated that endothelial cells in our newly developed 96-wells fluidic system respond to fluid flow-induced shear stress by aligning along the flow direction and increasing the levels of KLF2 and KLF4. We further demonstrate that our 96-wells fluidic system allows for efficient gene knock-down compatible with automated liquid handling for high-throughput screening platforms. Overall, we propose that this modular 96-well fluidic system is an excellent platform to perform genome-wide and/or drug screenings to identify the molecular mechanisms involved in the responses of endothelial cells to high wall shear stress.

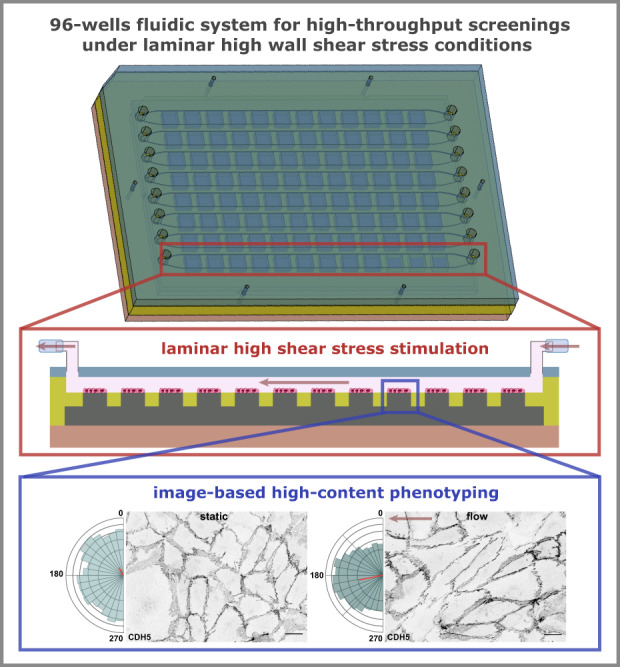

## Introduction

Endothelial cells (ECs), the cells lining the interior of blood vessels, are permanently under the mechanical forces exerted by blood flow. Wall shear stress (WSS), the frictional force parallel to the vessel wall, exerts an influence in EC biology^1^. In fact, ECs are able to sense WSS and convert it into a chemical response—mechanotransduction^2^. In response to blood flow, ECs reorganize and orient their cytoskeleton elongating and aligning parallel to the flow direction^3^, altering protein localization at the cell membrane^4–6^, and modifying the gene expression profile^7^. A striking example of mechanotransduction in ECs is the upregulation of the Krüpple-like factors (KLF)2 and KLF4 transcription factors^8,9^ and flow-migration coupling, which characterizes the polarization and migration of ECs against the flow direction^10–13^.

The hierarchical structure of blood vessels depends on the ability of ECs to sense blood flow. ECs discriminate levels of shear stress, and migrate from low-flow segments towards high-flow segments, leading to the regression of poorly perfused vessels^10,14^. Angiogenic ECs (tip cells) migrate against the flow direction toward emerging arteries, contributing to their growth^15,16^. Loss of ALK1-signaling in ECs impairs flow-migration coupling leading to EC accumulation in capillaries, which results in the formation of arteriovenous malformations^13^. In addition, blood flow and chemoattractants compete to establish the transition between sprouting and remodeling programs^12^. Furthermore, the presence of disturbed and unstable flow in vessel bifurcations is associated with a pro-inflammatory stimulus promoting atherosclerosis, whilst laminar flow is a strong pro-quiescence and anti-inflammatory stimulus^17,18^. These effects are linked to the flow-dependent regulation of KLF2/4, NO production, and active NF-*k*B levels^8,19,20^. Confirming the relevance of KLF factors, Sangwung et al. reported that EC-specific deletion of KLF2 and KLF4 is lethal^21^. Histological analysis of mutant mice revealed the presence of hemorrhages in small vessels of the brain, lungs, and heart, alongside with stroke-like symptoms. These results confirmed that KLF2 and KLF4 are fundamental in ECs to maintain normal EC physiology and blood vessel integrity^21^.

Not surprisingly, defective endothelial responses to blood flow are associated with vascular pathology. In regions of disturbed flow prone to atherosclerosis development was shown that inflammation leads to aberrant EC response to blood flow such as defects in EC polarity^22,23^. EC-specific knock-out (KO) mice for Par-3 showed that Par-3 regulates the establishment of EC polarity to the flow axis promoting the expression of pro-inflammatory molecules^24^. In these regions and regions of vessel bifurcation, there is an accumulation of plaques due to inflammation which can lead to vessel narrowing and consequent rupture of these plaques may result in stroke^25^.

Also, it was shown in vivo that absence of endoglin in ECs compromised their ability to sense and respond to blood flow. In this context, it was observed that ECs accumulate in the vessels and fail to align within the vessel in response to flow due to an increase in EC surface and aberrant EC shape resulting in shunt formation and arteriovenous malformations development^26,27^. Furthermore, the impairment of mechanotransduction mediated by Smad1/5 downstream of Alk1/Eng leads to vessel enlargement promoting shunt formation^28^, through the regulation of connexin-37 expression. Absence of connexin-37 impacts proper EC directional migration in response to blood flow, which contributes to vessel enlargement leading, ultimately, to shunt formation^29^.

Several cell surface protein complexes have been shown to be mechanosensitive to WSS, such as ion channels^30,31^, primary cilia^32,33^, focal adhesions^34,35^, and cell junctions^36,37^. For instance, the ion channel protein Piezo1 was shown to be required for EC alignment along flow direction. Moreover, Piezo1 EC-specific KO mice exhibited defects in pruning of the vascular network and ECs in arteries do not align with the flow direction^38^. Recently, the guidance receptor PlexinD1 was identified as being essential for EC alignment along flow direction and upregulation of *klf2* and *klf4* under laminar flow. When absent, it leads to the upregulation of pro-inflammatory genes^39^. The relevance of the junctional complex, composed of PECAM1, VE-cadherin, and VEGFR2, was also described in the morphological adaptation of ECs in response to flow^37^. The absence of PECAM1 and VE-cadherin impairs EC alignment along flow direction and impairment of integrin activation^37^. In fact, integrins are fundamental for EC flow response. For example, when integrin β1 is silenced in ECs, these cells fail to align in the aorta where blood flow is laminar and unidirectional^40^. Moreover, in vitro data showed that blocking integrin activation results in defects in focal adhesion formation, which ultimately decreases the ability of ECs to polarize against the flow direction^12^. Recently, RNAi screening performed by Xu et al. identified a new flow sensor, GPR68, with an important function for vessel physiology and pathophysiology. This newly identified flow sensor responds not only to laminar flow but also to disturbed flow, yet in low flow conditions. GPR68 is expressed in ECs from small diameter arteries of several organs and was shown to be involved in vessel remodeling by controlling vasodilation. In fact, mice depleted for GPR68 showed impaired flow-mediated outward remodeling in small arteries suggesting this flow sensor is important to regulate remodeling of the arterial endothelium^41^.

Although the importance of several proteins has been studied in EC flow sensing, we still miss a clear understanding of the molecular mechanism that regulates the diversity of EC flow responses. To do a systematic and unbiased discovery program of the molecular mechanisms regulating EC flow responses, there is a need for fluidic systems allowing genome-wide high-throughput screenings under high flow conditions.

Several studies have been using microfluidic channels to study biomolecular interactions, either cell-substrate interactions or protein–protein interactions under perfusion conditions. By functionalizing the channel substrate with different adhesion molecules is possible to study the adhesion of cells to a given substrate. In addition, by playing with microchannels geometries is possible to vary the shear force applied in the system in parallel in the same experimental setup^42^. Several techniques have been developed to study protein–protein interaction, such as Force spectrometry or dielectrophoresis. In both techniques microchannels are functionalized with an antibody/receptor of interest and beads coated with ligands/antigens are introduced in the channels^43–46^. In force spectrometry, pressure driven flow is applied and is possible to measure the strength of receptor-ligand bond^43^. Dielectrophoresis in conjugation with shear force allows to identify the interactions by quantifying the beads eluted from the channel bonded to the proteins from the channel surface^44–46^. Dielectrophoresis can be also used in combination with force spectrometry allowing to quantify the strength of the protein–protein interactions^45^.

Moreover, a variety of platforms to study the effects of shear stress in ECs have been developed, yet most have limitations that restrain their usage. Cone-plate devices have the ability to perform high-throughput small interference RNA (siRNA) screenings, yet in these platforms the shear stress profile varies inside the well, hindering precise analyses of EC flow responses^47^. Microfluidic customized cell culture channels have become increasingly popular in the last decade^48^. For instance, Sinha et al. developed a device that combines both surface strain and shear stress on the cells, with a flow array component containing multiple channels. In this device, the shape of the channels enables having different levels of shear stress and the cells are seeded on top of pillars. Since flow channels are assembled before the cell culture, siRNA transfection in a high-throughput manner is not possible using this setup^49,50^. In a different platform, a 384-wells system was designed by Xu and colleagues, to perform high-throughput RNAi screening under flow conditions. In this system, 384 flat-headed pistons, driven by an acoustic transducer, were used to create shear stress in each well by moving up and down the piston at a given amplitude^41^. Although it allows having a high-throughput system to perform siRNA screening, the movement of the piston generates disturbed fluid motion with an oscillatory pattern and non-uniform laminar flow. More recently, Wei and colleagues reported a new system to perfuse regular 96-wells plates. The advantage of this system is the possibility of using regular cell culture plates to do the cell culture and siRNA transfection in a high-throughput and automated manner.

However, the medium that flows in the wells is turbulent in its profile and creates a gradient of very low shear stress generating a diversity of flow response^51^.

**Fig. 1:**
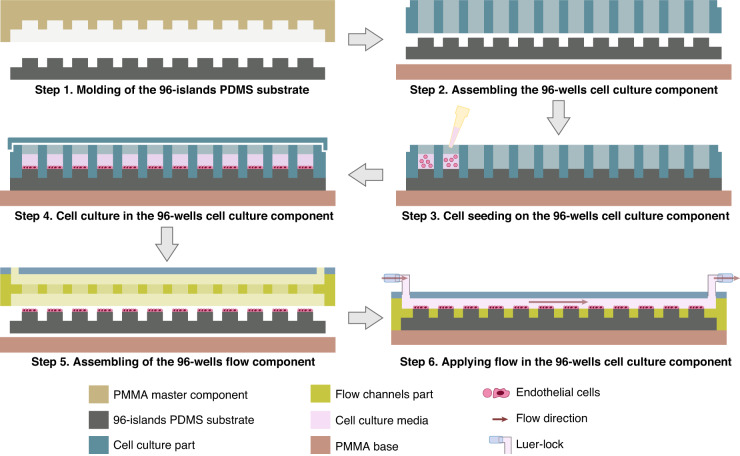
Conceptual 2D representation and workflow of the 96-wells fluidic system. Step 1: The 96-islands PDMS substrate is obtained through PDMS molding by using the PMMA 96-wells master. Step 2: The 96-islands PDMS substrate and the 96-well cell culture part (made of PMMA) are sterilized and assembled by inserting the 96-islands PDMS substrate into the 96-well cell culture part. The parts are bond together by using commercial metal binder 1.6 inches wide paper clamps on the sides. This creates the 96-wells cell culture component. Step 3: Each well of the 96-islands PDMS substrate is coated with the ECM of interest followed by seeding of cells on each PDMS well. Step 4: Adherent cells will grow forming a monolayer. A lid of a regular cell culture plate is used in order to maintain aseptic cell culture conditions. At this step, cell manipulation can be performed, such as siRNA transfection. Cell monolayers will remain in the 96-well cell culture component until ready to be exposed to flow. Step 5: The 96-wells cell culture component, with the cells, is disassembled by removing the lid and the 96-well culture part. The flow channels part, containing the inlet/outlet and channels part, is then inserted on top of the 96-islands PDMS substrate and the PMMA base to create the 96-wells flow component. The channels part contains 96 cavities of the same height as the PDMS islands. When assembled, the 96-wells flow component creates 8 linear and independent flow channels of 500 µm height. Step 6: The 96-wells flow component is ready to be coupled to a fluidic system via elbow luer connectors, or equivalent, allowing perfusion of cell culture media at the desired flow rate

In this paper, we report the development of a modular 96-wells fluidic system, based on the standard configuration of cell culture plates, to perform high-throughput siRNA screenings under physiological levels of shear stress and laminar fluid flow profile (Fig. 1). Our platform will be key to investigate the molecular mechanisms that regulate EC flow responses.

## Materials and methods

### 96-wells fluidic system design and fabrication

The 96-wells fluidic system was designed using Autodesk AutoCAD software. Dimensions are based on a standard 96-wells cell culture plate to potentiate the usage of automated platforms for cell culture and transfection in future applications. After initial prototyping, the production of solid parts in poly(methyl methacrylate) (PMMA) using 3D CNC milling processing was outsourced to ZEG-MED, Poland. This allowed us to obtain higher resolution and higher refinement of finishing and bonding. The polydimethylsiloxane (PDMS) parts were fabricated by soft-lithography^52^ from masters in PMMA.

**Fig. 2:**
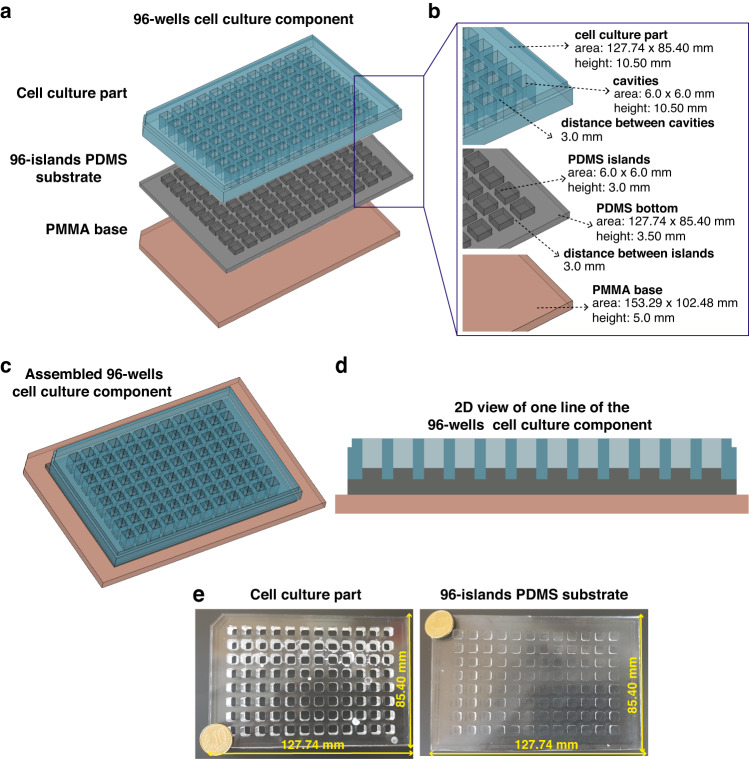
Design and dimensions of the 96-wells cell culture component. **a** The 96-wells cell culture component is composed by a cell culture part with 96 cavities made of PMMA, where the PDMS islands will enter creating a cell culture well, a 96-islands PDMS substrate and a PMMA base. The cell culture part has a small retracted offset on the top edges, allowing the use of a lid to maintain the aseptic cell culture conditions. **b** Dimensions are presented for all the parts and components together with a close-up to show the relevant features and measures of the different designs. **c** The 3 components are connected to create the 96-wells cell culture component, ready to be used for cell seeding and cell culture conditions. **d** 2D transversal view of a representative line of the assembled 96-wells cell culture component. **e** Representative images and relevant dimensions of the cell culture part (left) and the 96-islands PDMS substrate (right)

**Fig. 3:**
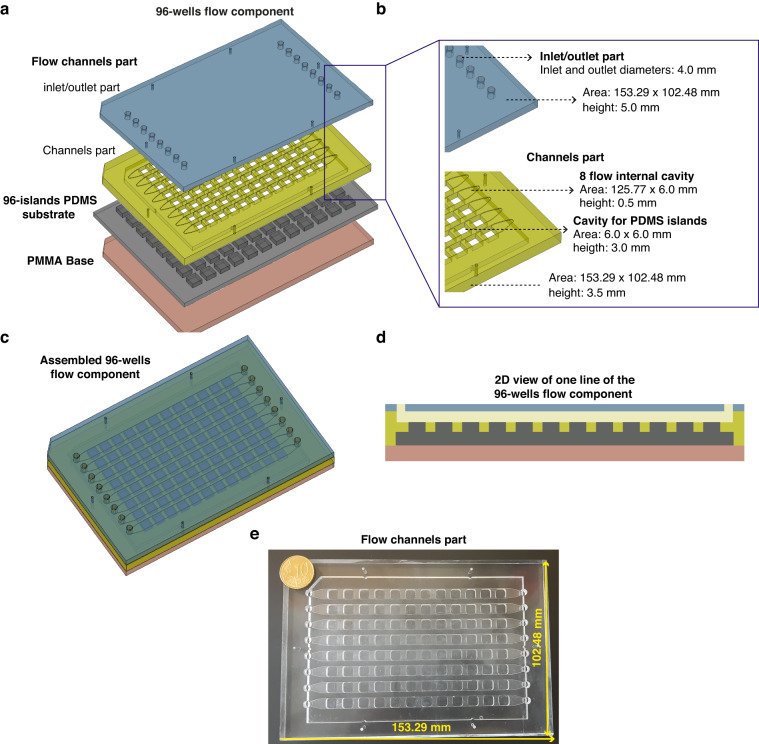
Design and dimensions of the 96-wells flow component. **a** The 96-wells flow component is composed of a flow channels part made of PMMA a 96-islands PDMS substrate and a PMMA base. The flow channels part is composed 8 flow channels, with respective inlets and outlets, which start and end with a smaller width adjusted to the inlet/outlet inner diameter. The flow channels part was generated by binding together, by thermal diffusion, a top inlet/outlet part (a PMMA plate containing inlet and outlets) with a channels part (a PMMA structure that contains a linear flow channel of 500 µm height and 96 cavities fitting the PDMS islands). **b** Dimensions are presented for all the parts and components together with a close-up to show the relevant features and measures of the different designs. **c** The 3 components are connected to create the 96-wells flow component, ready to be used for high-flow experiments. **d** 2D transversal view of a representative flow channel of the assembled 96-wells flow component. **e** Representative image and relevant dimensions of the flow channels part

The 96-wells fluidic system (Fig. 1) requires 3 different components: the PMMA master component (Supp. Fig. 1), the 96-wells cell culture component (Fig. 2), and the 96-wells flow component (Fig. 3). Each one is described below in more detail.

The PMMA master component (Supp. Fig. 1) is used to create the 96-islands PDMS substrate required for the 96-islands cell culture component and the 96-wells flow component, to which EC adhere (Fig. 2). The PMMA master component is composed of two PMMA modules (Supp. Fig. 1a). The first module is a smooth PMMA plate (153.29 × 102.48 × 5.0 mm) that acts as a lead for the PDMS islands, termed PMMA plate (Supp. Fig. 1a, b). The second module, termed PMMA 96-wells master, is a PMMA structure (153.29 × 102.48 × 6.5 mm) with an internal cavity (127.74 × 85.40 × 3.5 mm), which will create the bottom of the 96-islands PDMS substrate. The base of the internal cavity contains 96-wells (6.0 × 6.0 × 3.0 mm) spaced by 3 mm from each other, which will give rise to the PDMS islands of the 96-islands PDMS substrate (Supp. Fig. 1a, b). The PMMA plate and the PMMA 96-wells master were bonded by thermal diffusion in order to create a single component, the PMMA master component (Supp. Fig. 1c). PDMS filling of the PMMA master will create the 96-islands PDMS substrate defining 2 height steps: a first to define the flat bottom, with 3.5 mm height, and a second for 96 islands of 6.0 × 6.0 × 3.0 mm.

The 96-wells cell culture component (Fig. 2) is used for cell culture and cell transfection. This component is composed of a cell culture part, a 96-islands PDMS substrate and a PMMA base (Fig. 2a, b). The cell culture part is a PMMA case (127.74 × 85.40 × 10.50 mm) that encloses 96 cavities (6.0 × 6.0 × 10.5 mm) drilled throughout the thickness of the plate, and spaced from each other by 3 mm (Fig. 2a, b). This part will insert into the 96-islands PDMS substrate, creating 96-wells with 7.5 mm of height (Fig. 2c, d). The top edges of the PMMA-base cell culture part have a small retracted offset in order to use a lid of a regular cell culture plate in order to maintain aseptic cell culture conditions (Figs. 1 and 2c, d). The 96-islands PDMS substrate is generated by the PDMS molding using the PMMA master component (Supp. Fig. 1). The PMMA base (153.29 × 102.48 × 5.0 mm) is a solid structure and is designed to provide support to the 96-wells cell culture component, and the 96-wells flow component (Fig. 2a, b). The 96-wells cell culture component is assembled together by inserting the cell culture part into a 96-islands PDMS substrate on top of the PMMA base (Fig. 2c, d). Representative pictures of the cell culture part and the 96-islands PDMS substrate are shown in Fig. 2e.

The 96-wells flow component (Fig. 3) is composed of three different PMMA parts, the flow channels part, the 96-islands PDMS substrate and the PMMA base (Fig. 3a, b). The 96-islands PDMS substrate and the PMMA base are the same structures defined in the previous paragraphs. The flow channels part is built of two different parts (the inlet/outlet part and the channels part) that were bonded together by thermal diffusion (Fig. 3a, b). The flow channels part (153.29 × 102.48 × 6.5 mm) contains 8 long channels with 0.5 mm of height (Fig. 3a). The inlet/outlet part (153.29 × 102.48 × 5.0 mm) is a PMMA case that contains 8 inlets and 8 outlets of the channels with a diameter of 4 mm each (Fig. 3b). The channels part is a PMMA structure (153.29 × 102.48 × 3.5 mm) with 8 flow internal cavities (125.77 × 6.0 × 0.5 mm), which will create individual flow channels upon assembly of the 96-wells flow component. The base of the channels part encloses as well 96 cavities (6.0 × 6.0 × 3.0 mm), drilled throughout the thickness of the structure and spaced from each other by 3.0 mm (Fig. 3a, b). This part will insert into the 96-islands PDMS substrate, creating 8 channels of 0.5 mm of height comprising 12 PDMS islands, which will be levelled to the same height (Fig. 1, Fig. 3c, d). This design allows the generation of a linear flow channel, with a constant cross-section. Our design has one additional particularity. The flow channels start and end with a smaller width adjusted to the inlet/outlet inner diameter (width of 4.0 mm) (Fig. 3b). This creates a stabilizing volume of media, prior to the PDMS islands. We utilize the same PMMA base, described in the 96-well cell culture component for cell culture, to give mechanical support to the system. The flow channels part is assembled with the 96-islands PDMS substrate and the PMMA base to create the 96-wells flow component (Fig. 3c). We used commercial metal binder 1.6 inches wide paper clamps to hold all the components tightly together in order to avoid leakage. A representative picture of the channels part is shown in Fig. 3e.

### PDMS preparation and molding

To prepare the PDMS, we used the PMMA master component. It is needed to flip the PMMA mold upside down in order to pour the PDMS. The 96-islands PDMS substrate was prepared by mixing silicone elastomer with curing agent (Sylgard 184 kit, Dow 101697) in a 10:1 ratio (%w/w), The mixture was degassed in vacuum chamber for 30 min at RT. Next, PDMS was poured into the master and another step of degassing (30 min at RT) was performed to remove bubbles formed. The PDMS was cured in an oven at 75 °C for 2 h, and particular attention was given to the levelling of the master and mold. After cross-linking, the PDMS was de-molded and ready to be used.

### Device assembling

Prior to assembling the device, all components were sterilized with 70% EtOH and UV light in a laminar flow chamber for 15 min. All essential steps of the procedure are depicted in Fig. 1. Then the 96-islands PDMS substrate was assembled with the cell culture part with a set of commercial metal binder 1.6 inches wide paper clamps to bond all components together. Before seeding the cells, PDMS islands were incubated with 10 µg/mL of fibronectin (diluted in PBS) for 1 h, at RT, to ensure cell adhesion. After removal of fibronectin, PDMS islands were washed twice with PBS followed by cell seeding. During cell culture, the assembly is covered with a standard lid over to secure sterility.

For experiments under flow, the 96-wells cell culture component was carefully disassembled. For this, metal binder 1.6 inches wide paper clamps are removed and the cell culture part is removed from the 96-island PDMS substrate. Then, the flow channels part is inserted carefully into the 96-island PDMS substrate to create the 96-well flow channels component. Since the 96-island PDMS substrate is not rigid, commercial metal binder 1.6 inches wide paper clamps were used again to compress the flow channels part together with the PMMA base to keep the system stable and tightly sealed. After assembly, an initial volume of cell culture medium was carefully introduced in the channels with the help of a syringe, prior to tubing connects for perfusion experiments.

### Flow on the 96-well fluidic system

To apply flow in 96-wells flow component, the channels are filled with flow medium - Leibovitz L15 media (LTI 21083-027, Life Technologies) supplemented with EGM-2 SingleQuotsTM (CC-4176, Lonza) and 1% penicillin/streptomycin (#15140122, Gibco), and the system is placed under culture conditions for 30 min to stabilize. After, each flow channel in the 96-well fluidic plate is connected to a peristaltic pump (Gilson Minipuls3), with independent feeding per each flow channel, ensuring the continuous laminar flow during the duration of each experiment. The shear stress applied in the system was 7 dyn/cm^2^ (0.7 Pa). After 4 h, the cells were fixed for immunofluorescence or used to perform RNA extraction.

### Computational fluidic dynamics simulation

To assess and better understand the behavior of fluid dynamics in the 96-well flow component, we employed a finite elements model for computational simulations using COMSOL Multiphysics software. We used the fluid flow module, specifically for the single-phase fluid in the laminar flow regime, governed by the Navier-Stokes equation. The study was stationary, water defined as the equivalent fluid for cell culture media and the flow used was incompressible. The density and dynamic viscosity were defined as of the water, and the walls were defined as no-slip wall. A CAD file with only 1 channel (comprising 12 islands) was imported to COMSOL (Supp. Fig. 2). Then a physics-controlled mesh using a fine element size was built and the results of the simulation were computed and plotted in terms of velocity, pressure and shear stress were analyzed for an initial velocity of 0.08 m/s.

### Cell culture

Human umbilical vein endothelial cells (HUVECs, C2519A, Lonza) were routinely cultured following the manufacturer’s guidelines, in filter-cap T75 flasks Nunclon ∆ surface treatment (VWR International, LLC) and cultured at 37 °C and 5% CO_2_ to ensure a stable environment for optimal cell growth. HUVECs were cultured with complete medium [EGM-2 Bulletkit (CC-3162, Lonza) supplemented with 1% penicillin/streptomycin (#15140122, Gibco)]. When passaging cells for experiments, cells were washed twice in sterile PBS (137 mM NaCl, 2.7 mM KCl, 4.3 mM Na_2_HPO_4_, 1.47 mM KH_2_PO_4_, pH 7.4). Then, cells were incubated for 3-5 min in TrypLE Express (#12605-028, Gibco) at 37 °C, 5% CO_2_. When 95% of the cells detached, complete medium was added to each flask to inhibit the activity of the TrypLE Express and the cell suspension was transferred to a falcon tube. HUVECs were then centrifuged at 700 rpm for 5 min at RT and the pellet was resuspended in fresh complete medium. The cell concentration present in the suspension was determined using a Neubauer Cell Counting Chamber (Hirschmann EM Techcolor). The cells were then seeded on PDMS islands at 1.8 × 10^5^–3 × 10^6^ cells/mL, depending on the experimental condition and placed in the incubator at 37 °C, 5% CO_2_. All experiments with HUVECs were performed between passages 3 and 6.

### siRNA transfection

To silence the expression of genes of interest, a set of ON-TARGET human siRNAs against CTNNA1 (Horizon Discovery, J-010505-06), CDH5 (Horizon Discovery, J-003641-07) or untargeting control were used (Horizon Discovery, D-001810-01), using previously defined conditions^53,54^. Briefly, HUVECs were seeded the day before the transfection at a concentration of 1.8 × 10^5^ cells/mL to reach 60–70% confluence on the day of the transfection. Then, cells were transfected with 25 nM of siRNA using Dharmafect 1 reagent (Horizon Discovery) following the Dharmacon siRNA transfection protocol. The cell culture medium was replaced 24 h after transfection by fresh complete medium and cells were kept under culture conditions up until 72 h post-transfection and then processed for further experiments.

### Immunofluorescence on PDMS islands

To perform immunofluorescence, HUVECs were fixed in 1% paraformaldehyde (PFA) supplemented with 1 M MgCl_2_ and 1 M CaCl_2_ (1 μL/2 mL) in PBS for 30 min at room temperature (RT). Then, cells were washed with 1X PBS to remove the remaining PFA followed by blocking and permeabilization of cells with a blocking solution containing 3% BSA in PBS-T (PBS with 0.1% Triton X-100) for 30 min at RT. After, cells were incubated with the appropriate primary antibodies for 2 h, at RT, diluted in the blocking solution (anti-VE-cadherin, R&D - AF938, 1:50; anti-αCatenin, Sigma-Aldrich - C2081, 1:200; anti-KLF4, R&D - AF3640, 1:200; anti-Golph4, Abcam-ab28049) and washed 3 × 15 min in PBS-T. Next, cells were incubated in blocking solution containing the appropriated secondary fluorophore-conjugated antibodies for 1 h at RT in the dark (Donkey anti-goat Alexa 647, Thermo Fisher Scientific - A21447, 1:400; Donkey anti-rabbit Alexa 568, Thermo Fisher Scientific - A10042, 1:400) followed again by 3 washes of 15 min in PBS-T. Finally, cells were incubated with 1x DAPI (Molecular Probes by Life Technologies) diluted in PBS for 5 min in the dark, followed by 3 washes with PBS. PDMS islands were then mounted with a glass coverslip and using Mowiol DABCO (Sigma-Aldrich). Images were acquired using a confocal Laser Point-Scanning Microscope 880 (Zeiss) equipped with a Plan-Apochromat DIC 63x NA 1.40 oil objective and the Zen black software. To quantify the KLF4 nuclear intensity, 5 images per PDMS island were acquired. The mean fluorescence intensity was measured using the FIJI software. Briefly, the DAPI channel was used as a reference to segment and select the nucleus as the region of interest (ROI) by setting a threshold. After, in the KLF4 channel, using the commands ROI to manager and analyze particles, we measured the mean intensity fluorescence of KLF4 in static and flow conditions, dictated by the ROIs defined in the DAPI channel.

### Quantification of HUVEC’s alignment

For quantitative analyses, 10 images per slide were acquired using a Zeiss Axiovert 200 inverted microscope (Carl Zeiss MicroImaging) equipped with the Metamorph software with an EC Plan-NeoFluar 40x NA 0.75 dry objective. To quantify cell and nuclei alignment, HUVECs under static and flow conditions were fixed and immunofluorescence was performed, as described previously. An anti-VE-cadherin antibody (R&D - AF938, 1:50) was used to label adherens junctions to mark cell outline and DAPI (Molecular Probes by Life Technologies) was used to label the nuclei of HUVECs. Images were acquired using a confocal Laser Point-Scanning Microscope 880 (Zeiss), equipped with a C-Apochromat Corr 40x NA 1.20 water objective and the Zen black software. To analyze EC alignment, 2 images per PDMS island were acquired using the tile scan mode (2 × 2). Then a maximum projection along z-stack was performed on all microscopic images by using Fiji. Images were segmented using the deep learning algorithm Cellpose, using the VE-cadherin as a proxy for the cell outline and DAPI for nuclei staining. Using scikit-image (python) and ellipse fit was performed to the shape of each cell and nuclei, respectively (Supp. Fig. 3). The angle of the major axis of that ellipse with the x-axis, representing the flow direction, serves as a readout for cell shape and nuclei orientation. An angular histogram showing the angle distribution was then generated. Circular statistics were performed according to the Circular Statistic Toolbox^55^. Note that cell shape and nuclei orientation angles are referred to as axial data, meaning that all orientation angles ${\alpha }_{i},i=1,...,N$ take values between 0 and 180 degrees. Here, any angle $\,{\alpha }_{i}$ is identified with its opposite ${\alpha }_{i}+$180, thus we do not distinguish the front and back of the cell (or nucleus). The axial orientation data were converted to unimodal data by doubling all values ${\theta }_{i}=2{\alpha }_{i}\,$. The mean direction was computed from $\bar{\alpha \,}=\frac{1}{2}\bar{\theta }$, where $\bar{\theta }$ is the common circular mean of the unimodal data ${\theta }_{i}$. Similarly, the polarity index (PI) was calculated as the length of the mean resultant vector of unimodal values ${\theta }_{i}$ and is given by:

$${PI}=\sqrt{{\left(\frac{1}{N}\mathop{\sum }\limits_{i=1}^{N}\cos {\theta }_{i}\right)}^{2}+{\left(\frac{1}{N}\mathop{\sum }\limits_{i=1}^{N}\sin {\theta }_{i}\right)}^{2}}$$

The PI value varies between 0 and 1 and indicates how much the distribution is concentrated around the mean. A value of PI close to 1 implies that the data are concentrated around the mean direction, while a value close to 0 suggests that the data are evenly distributed or random. In summary, the PI indicates the collective orientation strength of the cell monolayer. All scripts are available at https://github.com/polarityjam, 10.5281/zenodo.8317234.

### RNA extraction and cDNA production

For RNA collection, after disassembling the 96-wells flow component, HUVECs were detached from the 96-islands PDMS substrate using TripLE Express (Thermo Fisher Scientific). For each condition, cells from 3 PDMS islands were collected and pooled together in a single RNase-free 1.5 mL tube and centrifuged for 5 min at 1000 rpm. After discarding the supernatant, the pellet was resuspended in Trizol and incubated for 10 min at RT. After, chloroform (Merck Millipore) was added. The tubes were shaken for 30 s and incubated at RT for 5 min and centrifuged for 15 min. Afterward, the upper aqueous phase was carefully transferred to a new tube. To precipitate the RNA, 1.5 µL of Glycogen (Sigma) and 1/10 of the volume of the sample of 3 M sodium acetate were added to each tube. After vortexing, 1 volume of isopropanol (VWR) was added to each tube, following by vortexing, and tubes were then incubated 15 min at RT. After, tubes were centrifuged for 8 min and the supernatant was discarded. The pellets were washed with 1 mL of 70% ethanol (VWR), the tubes were centrifuged for 5 min, and the supernatants were discarded. The pellets were allowed to dry at RT and then resuspended with 20 µL of RNase-free water (Sigma) and kept on ice, followed by RNA quantification using Thermo Scientific NanoDrop 2000. After quantification, samples were treated with RNase-free DNase I (Roche) for 20 min at 30 °C. 1 volume of phenol-chloroform-isoamyl alcohol mixture (Amresco) was added to the samples, to inactivate the DNase I and to purify the RNA. After centrifugation for 10 min, the upper aqueous phase was transferred for a new eppendorf, 1 volume of chloroform was added and tubes were centrifuged for 10 min. The upper aqueous phase was transferred to a new eppendorf and the precipitation and washing steps were repeated. After drying, the pellets were resuspended in 15 µL of RNase-free water, kept on ice and quantified using Thermo Scientific NanoDrop 2000. A fraction of the purified RNA (between 84 and 122 ng) was used to produce cDNA, using the High-Capacity RNA-to-cDNA Kit (Applied Biosystems), following the manufacturers’ protocol. The produced cDNA was stored at −20 °C and used for real-time quantitative PCR (RT-qPCR).

### Real-time quantitative PCR

To quantify the gene expression in static vs flow conditions in the 96-well fluidic plate, we performed RT-qPCR (Table 1). In every RT-qPCR run, a standard curve was obtained for each primer pair alongside each sample, by mixing cDNA from all the conditions tested and then three different dilutions were made (1:10, 1:25; 1:50). For each reaction a mix of 7 µL of Power SYBR Green PCR Master Mix, 0.3 µL of primers pair (final concentration of 100 nM), 2 µL cDNA and 4.85 µL of RNAse-free water were prepared to result in a final volume of 14 µL per well. The RT-qPCR reaction was performed in the Applied Biosystems VIIA 7 Real-Time PCR system using the standard protocol. The results were analyzed in the QuantStudio Real-time PCR Software (Applied Biosystems). The expression levels of each sample duplicate were then normalized to GAPDH, and the Livak Method (2-ΔΔCq) was used to calculate the relative changes in the gene expression. The graphs were plotted using Graph Pad Prism 8 software.

**Table 1:** siRNA sequences used in KD experiments in the 96-well fluidic system

siRNA	siRNA target sequences	Vendor/Catalog #
siCtl	UGGUUUACAUGUCGACUAA	Dharmacon/D-001810-01
siCdh5	GAGCCCAGGUCAUUAUCAA	Dharmacon/J-003641-07
siCtnna1	GAUGGUAUCUUGAAGUUGA	Dharmacon/J-010505-06

Details of ON-TARGET siRNA target sequences catalog number

### Polarity analysis

To quantify cell polarity, 10 images per slide were acquired using a Zeiss Axiovert 200 inverted microscope (Carl Zeiss MicroImaging) equipped with the Metamorph software with an EC Plan-NeoFluar 40x NA 0.75 dry objective. Images of HUVECs stained with Golgi (Golph4) and nuclear (DAPI) markers were processed in FIJI. Next, each set of images was imported and analyzed using a dedicated MATLAB script, published previously^12,14^. Succinctly, the script segments individually the fluorescent signal of the Golgi complex and nuclear stainings, identifies the centroid of each organelle and calculates a vector connecting the centroids of the nucleus and its corresponding Golgi apparatus. The Golgi-nucleus assignment is done automatically by minimizing the distance between all possible pairs. The polarity of each cell was described as the angle between the vector and the slide axis. Angular histograms show the distribution of the angles. Circular statistic was performed using Circular Statistic Toolbox^55^. The polarity index (PI) was calculated as the length of the mean resultant vector for a given angular distribution and varies between 0 and 1, indicating the collective orientation strength of the cell monolayer. Values closer to 0 correspond to random distribution, while values closer to 1 indicate that the angle values are concentrated around the mean direction.

## Results

### Computational fluid dynamics

**Fig. 4:**
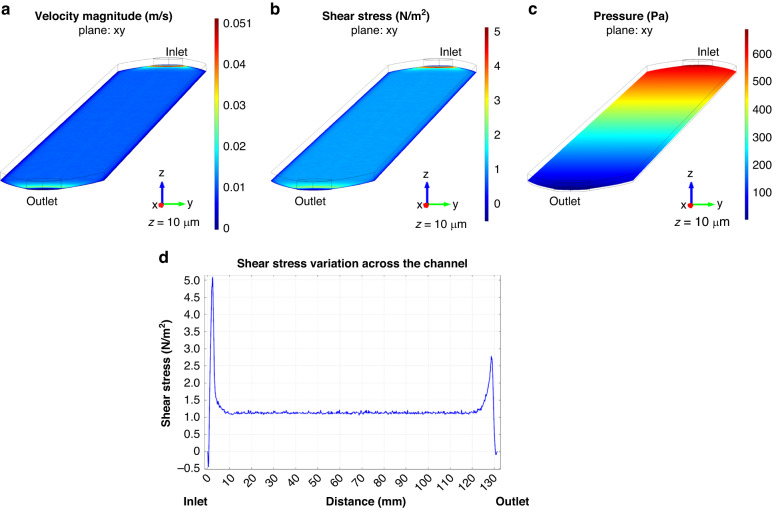
Computational fluidic dynamics in 96-well fluidic plate. **a** Simulation of the velocity magnitude (m/s) across the channel, in the xy plane at 10 µm distance above the bottom of the channel/well (z = 10 µm). **b** Simulation of the shear stress profile (N/m^2^) across the channel in the xy plane at 10 µm distance above the bottom of the channel/well (z = 10 µm). **c** Linear graph showing the variation of shear stress across the channel at 10 µm distance above the bottom of the channel/well (z = 10 µm). Gray squares represent the approximated location of the PDMS islands along the channel. **d** Simulation of the pressure profile (Pa) across the channel in the xy plane at 10 µm distance above the bottom of the channel/well (z = 10 µm)

Analysis of velocity, shear stress, and pressure profile was performed in a single representative longitudinal channel of the 96-well fluidic system, which includes 12-islands under perfusion (Supp. Fig. 2). The plot for velocity magnitude is extracted from the xy plane at the height of 10 µm from the bottom of the channel/well (z = 10 µm). This revealed a linear velocity profile with very small variations across the channel, with an increase in the velocity only near the inlet and outlet regions, mostly due to the smaller width narrowing of the channel in those regions (Fig. 4a). In addition, transversal and longitudinal cross sections of the velocity profile also showed that the velocity has a minimal variation across most of the channel. The velocity reaches a maximum in the center of the channel and a minimum value near the walls of the channel (Supp. Fig. 4a). Similarly, the shear stress profile in the xy plane, for z = 10 µm, showed a very small variation across the channel, with higher values near the inlet and outlet due to the smaller width of the channel (Fig. 4b, d). In agreement, transversal, and longitudinal cross-sections showed no differences across the channel in terms of the shear stress profile (Supp. Fig. 4b). The pressure profile presented, depicts the drop in the pressure, from the inlet to the outlet as expected for the flow regime simulated (Fig. 4c; Supp. Fig. 4c). Yet, this drop in pressure becomes negligible when considering the overall contribution from tubing, which was disregarded in the simulation. Thus, our simulations revealed minimal variations of velocity and shear stress profile across the channel area (in both x and y), which demonstrates high homogeneity per PDMS island along the same channel of the 96-wells flow component.

### HUVECs respond to flow in the 96-well fluidic plate

**Fig. 5:**
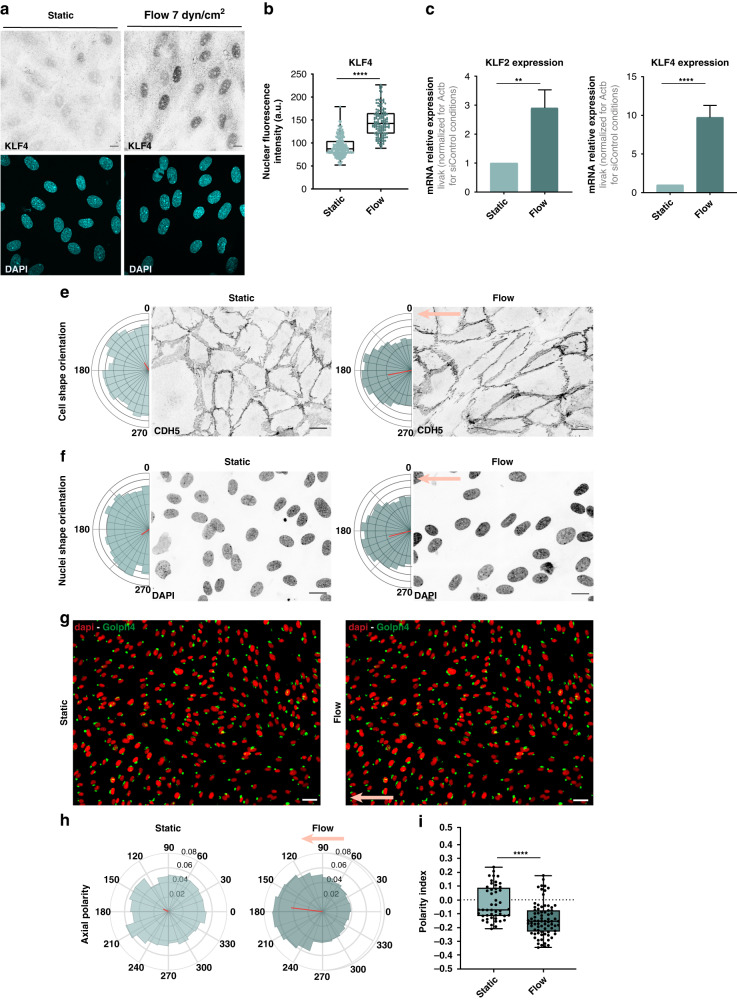
HUVECs respond to flow on the 96-well fluidic plate. **a** Fluorescence images for HUVECs under static and flow (7 dyn/cm^2^ of shear stress for 4 h) conditions in the 96-well fluidic plate labeled for KLF4 (gray) and for nuclei (DAPI, cyan). **b** Quantification of KLF4 nuclear fluorescence intensity in HUVECs under static or flow (7 dyn/cm^2^ of shear stress for 4 h). ****P < 0.0001 (Mann–Whitney test). Each point represents 1 cell. Box plot bars represent median, minimum and maximum values (*n* = 2). **c** Quantification of the mRNA relative expression of KLF2 or KLF4 by RT-qPCR in HUVECs under static and flow (7 dyn/cm^2^ of shear stress for 4 h) conditions in the 96-well fluidic plate. **P < 0.005; ****P < 0.0001 (unpaired Student´s t-test). Error bars indicate standard deviation (*n* = 3). **d** Angular histograms showing the distribution of EC shape orientation and representative images of ECs labeled with VE-cadherin in static or flow conditions (7 dyn/cm^2^ of shear stress for 4 h). Flow direction is right to left. Scale bar: 20 µm; *n* = 955 and 1478 cells for static and flow, respectively, from 3 different experiments. **e** Angular histograms showing the distribution of EC nuclei shape orientation and representative images of ECs nuclei labeled with DAPI in static or flow conditions (7 dyn/cm^2^ of shear stress for 4 h). Flow direction is right to left. Scale bar: 20 µm; *n* = 955 and 1478 cells for static and flow, respectively, from 3 different experiments. The light red arrow depicts the direction of flow. **f** Representative images of EC axial polarity orientation of HUVECs under static or flow conditions (7 dyn/cm^2^ of shear stress for 4 h). Flow direction is right to left. The light red arrow depicts the direction of flow. **g** Angular histograms showing the distribution of EC axial polarity orientation of HUVECs under static or flow conditions (7 dyn/cm^2^ of shear stress for 4 h). Flow direction is right to left. Scale bar: 50 µm; *n* = 1599 and 6145 cells for static and flow, respectively, from 2 different experiments. The light red arrow depicts the direction of flow. **h** Box plot bars represent median, minimum and maximum values (*n* = 2). Each point represents 1 field-of-view. ****P < 0.0001 (unpaired Mann–Whitney test). The light red arrow depicts the direction of flow

Next, we functionally tested the fluidic plate using ECs. KLF2 and KLF4 are transcription factors sensitive to shear stress and they regulate downstream flow-dependent transcriptional responses in ECs. Several studies showed upregulation of KLF2 and KLF4 in cells subjected to laminar and pulsatile shear stress but not in disturbed flow conditions^8,19,56^. In fact, KLF2 regulates around 46% of high flow-responsive genes (Parmar et al., 2006; Atkins and Jain, 2007), most of them being atheroprotective genes under laminar flow^8,57^. To validate the flow response of HUVECs in the 96-well fluidic system, we exposed ECs to a laminar and stable flow rate of 14 mL/min, corresponding to a shear stress of 7 dyn/cm^2^ (0.7 Pa). This value corresponds to an intermediate level of shear stress that ECs experience in veins in vivo^12,58,59^. This value is determined by the maximum value of flow rate before leakage starts occurring. Above this value of flow rate, and in our experimental conditions, we see leakage coming from inlets and outlets and sometimes also between flow lines. Keeping flow rates below this level prevents leakages. HUVECs were exposed to flow for 4 h. To assess EC flow response, we quantified the nuclear fluorescence intensity of KLF4 protein in similar conditions. We observed a significant increase in the expression of KLF4 in these cells under flow when compared to static conditions (Fig. 5a, b). Moreover, the increase in KLF4 was very homogenous across different wells, which confirms that the flow profile is homogeneous in each PDMS island along the same channel, as predicted by the computation fluid dynamics simulations (Fig. 4). To further confirm these results, KLF4 and KLF2 mRNA levels were assessed by RT-qPCR in HUVECs collected from individual channels in static or flow conditions. For both genes, we observed a statistically significant increase in their expression under flow in comparison to static conditions (Fig. 5c). These results demonstrate that ECs respond to flow in the 96-well fluidic system in a very homogenous way.

EC alignment and elongation along the flow direction has been observed in several studies in vivo and in vitro^5,23,60^. Also, ECs exposed to disturbed flow showed poor alignment and increased cell rounding^3,23^. To further validate the 96-well fluidic system, we evaluated the impact of fluid flow on EC shape orientation and alignment in comparison to static conditions. We measured HUVECs shape orientation and nuclei orientation using the polarity index (PI) as a readout for EC alignment, using Polarity Jam (reference 10.5281/zenodo.8317234). Polarity analysis of shape orientation of HUVECs exposed to flow for 4 h revealed that ECs showed alignment along the flow direction (PI = 0.224) in comparison to static conditions (PI = 0.0305) (Fig. 5d). Nuclei of HUVECs exposed to flow also aligned along the flow direction (PI = 0.198) when compared to nuclei of HUVECs under static conditions (PI = 0.0362) (Fig. 5e). The orientation of cell and nuclear shape of HUVECs along the flow direction confirms that ECs respond to flow in the 96-well system. Overall, these results confirm that the 96-well fluidic system enables the stimulation of EC with flow conditions and that ECs respond by increasing the expression of KLF genes and by orienting along the flow direction, which are indicative of a laminar and pulsatile flow regime.

Another well-described in vivo and in vitro ECs response to flow is the polarization against the flow direction. This polarization also known as front-rear polarity, or axial polarity, is given by the positioning of the Golgi or the centrosome and the nucleus, which established an axis of polarity (nucleus-to-Golgi axis)^61,62^. Axial polarity is positively correlated with the magnitude and levels of shear stress as shown in vivo and in vitro when HUVECs were exposed to different levels of shear stress for 4 h^12,14^. Thus, we accessed the polarization of ECs in our 96-wells flow component exposed to flow –7 dyn/cm^2^ for 4 h. Polarity analysis revealed that ECs polarize with the flow direction when cells are exposed to flow (PI = −0.143), as opposed to randomized polarization in static conditions (PI = −0.022) (Fig. 5f–h). These results further demonstrate that ECs can sense shear stress and respond to flow.

### HUVECs show high levels of KD efficiency in the 96-well fluidic system

**Fig. 6:**
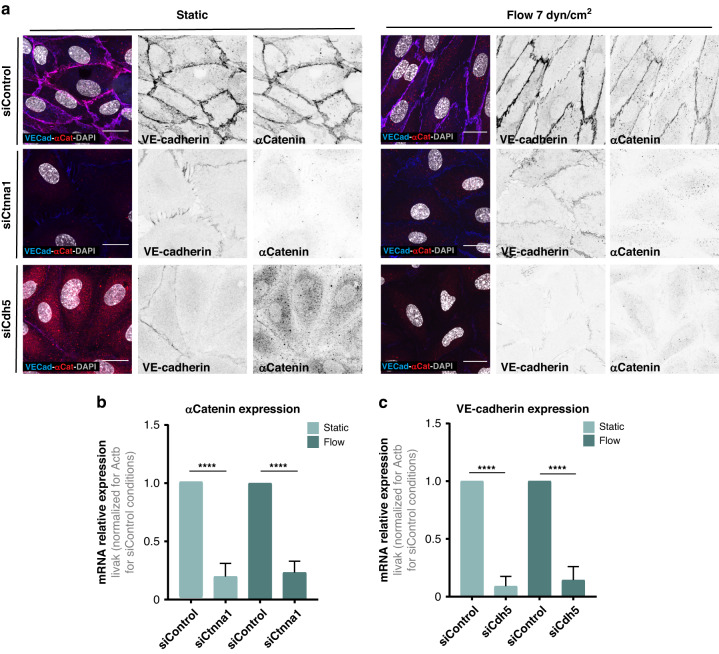
HUVECs showed good KD efficiency on the 96-well fluidic plate. **a** Fluorescence images of control (siControl) αCatenin- and VE-cadherin-depleted (siCtnna1, siCdh5, respectively) HUVECs under static and flow (7 dyn/cm^2^ of shear stress for 4 h) conditions. HUVECs were labeled for nuclei (DAPI, white), αCatenin (adherens junctions, red), and VE-cadherin (adherens junctions, blue). Black and withe images show VE-cadherin or aCatenin labeling. Scale bar: 20 µm. **b** Quantification of αCatenin mRNA relative expression by RT-qPCR in control (siControl) and aCatenin-depleted (siCtnna1) HUVECs in static and under flow (7 dyn/cm^2^ of shear stress for 4 h) conditions. Expression levels normalized to each control (siControl) condition. ****P < 0.0001 (one-way ANOVA with Tukey correction for multiple comparisons). Error bars indicate standard deviation (*n* = 3). **c** Quantification of VE-cadherin mRNA relative expression by RT-qPCR in control (siControl) and VE-cadherin-depleted (siCdh5) HUVECs in static and under flow (7 dyn/cm^2^ of shear stress for 4 h) conditions. Expression levels normalized to each siControl condition. ****P < 0.0001 (one-way ANOVA with Tukey correction for multiple comparisons). Error bars indicate standard deviation (*n* = 3)

Next, we validated that our 96-wells fluidic system is compatible with efficient gene silencing. We targeted two different genes coding for important proteins forming adherens junctions in ECs: *CTNNA1* and *CDH5* coding for αCatenin and VE-cadherin, respectively. We selected these two targets given the high selectivity of tools to quantitatively measure the efficiency of knock-down by immunofluorescence^12,54^. In addition, VE-cadherin, together with PECAM1 and VEGFR2, form an important flow-sensitive mechanosensor complex in ECs^12,37^. Absence of VE-cadherin affects EC flow response by impairing the alignment of ECs under flow^12,37^. We used previously validated siRNAs against *CTNNA1* and *CDH5*^12,54^. We assessed levels of αCatenin and VE-cadherin 72 h post-transfection in static and flow conditions. HUVECs transfected with siCTNNA1 showed complete abrogation of αCatenin from adherens junctions and a strong reduction in the expression of VE-cadherin both in static and flow-stimulated ECs. Similarly, siCDH5 transfected cells showed a dramatic reduction of both VE-cadherin and αCatenin expression in static and flow-stimulated cells (Fig. 6a). These observations were also confirmed and quantified by RT-qPCR, which clearly showed a statistically significant reduction of *CTNNA1* and *CDH5* in siCTNNA1 and siCDH5 conditions, both in static and flow conditions, respectively (Fig. 6b, c). These results confirm that our 96-wells fluidic system is suitable to perform high-throughput siRNA-based screenings under laminar high-flow conditions.

## Discussion

ECs in blood vessels are constantly subjected to changes in mechanical forces derived from pulsatile blood flow, circumferential stretch, vessel contraction, and trafficking of immune cells. Precise sensing and integration of these mechanical forces are essential to maintain vascular homeostasis^63,64^. However, the molecular mechanisms that regulate EC flow responses remain poorly understood.

Here we disclosed the development of a novel 96-wells fluidic system (Fig. 1) to perform image-based high-content screenings under high flow. The design of the 96-wells culture component allows cell seeding and any type of transfection prior to applying flow. In addition, the dimensions of this component and the distance between wells are similar to the ones from regular 96-wells cell culture plates, which allows the use of robotic platforms to perform all cell culture and transfections steps. The development of this 96-wells fluidic system was optimized in order to be compatible with automated platforms to perform cell culture and microscopy imaging acquisition. Yet, we did not test directly the performance of the 96-wells fluidic system plate in any specific automated system. By immunofluorescence and RT-qPCR, we validated the efficiency of siRNA transfection by abrogating the expression of two proteins of adherens junctions of ECs. Although we only validated this system regarding the siRNA transfection to KD proteins of interest, we expect that this platform could also be used with different reagents to perform KD or over-expression of proteins of interest, such as CRISPr-Cas9, plasmids, or viral vectors, or eventually to use drugs.

The flow response in ECs was further validated in the 96-well fluidic system. Our plate design allowed for the application of flow conditions on different 8 channels with independent shear stress conditions. In this system, each channel includes 12 islands that can be independently sampled. By applying physiological levels of wall shear stress, we observed an increase in the expression levels of both KLF2 and KLF4, two transcription factors sensitive to sheer stress levels^8,19,56^. In addition, we also observed the alignment of HUVECs and their nuclei with the flow direction, as well as significant polarization with flow direction. Altogether, these results provide strong evidence that ECs sense and respond to shear stress. These observations, together with the results obtained in the computational fluidic dynamics, support that ECs in the 96-wells fluidic system are under a laminar flow regime.

In conclusion, here we developed a fluidic platform that can be used to perform valuable high-content screenings for vascular biology under homogeneous laminar flow, using physiological levels of shear stress.

### Supplementary information

Supplementary Figures
