# Supplementary material for: A 96-wells fluidic system for high-throughput screenings under laminar high wall shear stress conditions
Source: Microsyst Nanoeng. 2023 Sep 15;9:114. doi: 10.1038/s41378-023-00589-x (PMC10504069; doi:10.1038/s41378-023-00589-x)
Supplement: Supplementary file 1 — Supplementary Figures [file 41378_2023_589_MOESM1_ESM.docx]

**Supplementary Figure 1**

**
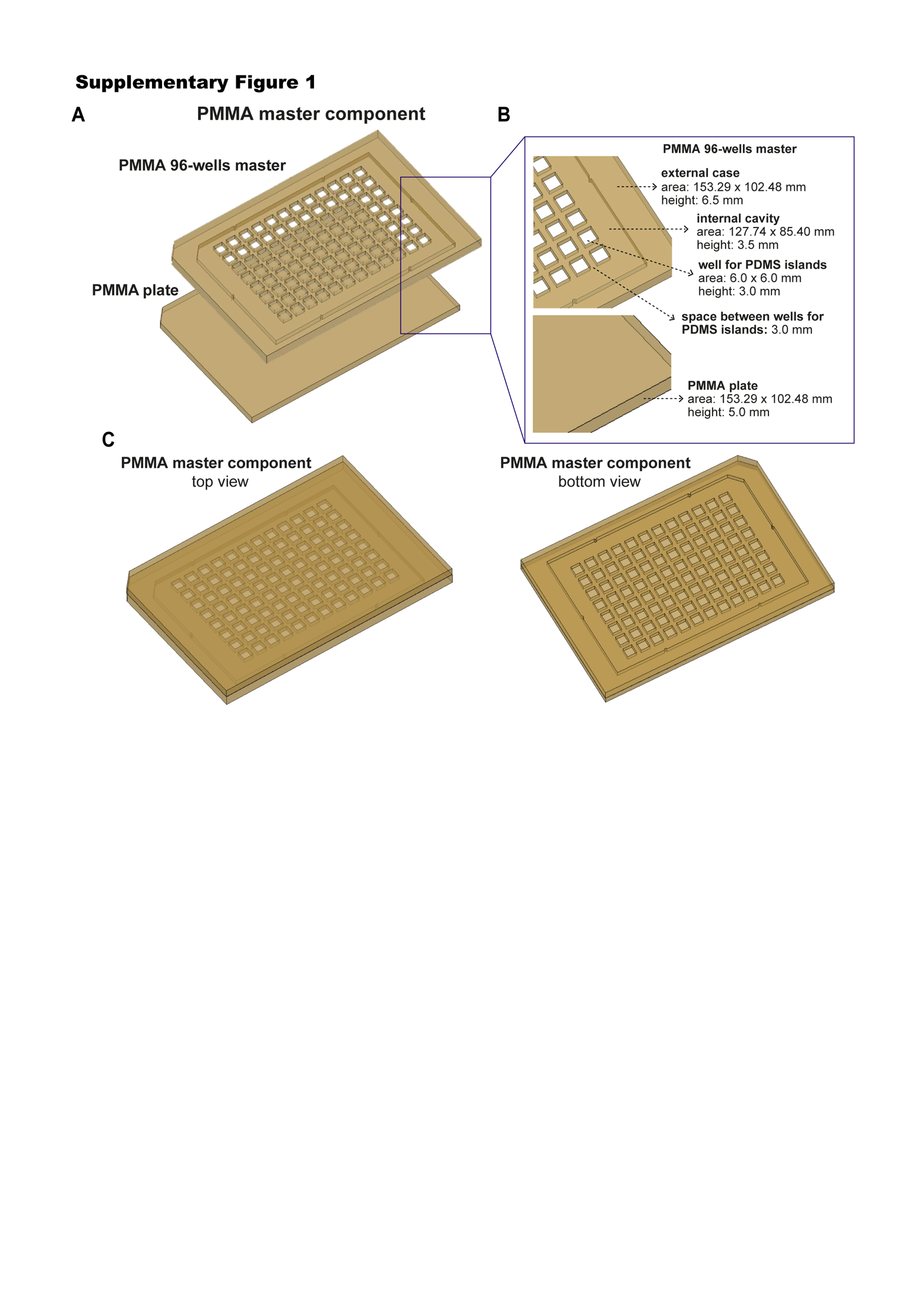
**

**Supplementary Figure 1. Design and dimensions of the PMMA master component.**

**(A)** The PMMA master component serve as a mold to generate the 96-islands PDMS substrate**.** The PMMA master component is composed by two different parts. The first one is a PMMA plate that creates smooth surfaces on PDMS islands. The second part is a PMMA 96-wells master that allows the generation of islands. **(B)** Close-up of the two parts of the PMMA master component with relevant dimensions. **(C)** The two PMMA parts were bound together, by thermal diffusion, to create the final PMMA master component seen by the top view (left) or its bottom view (right).

**Supplementary Figure 2**

**
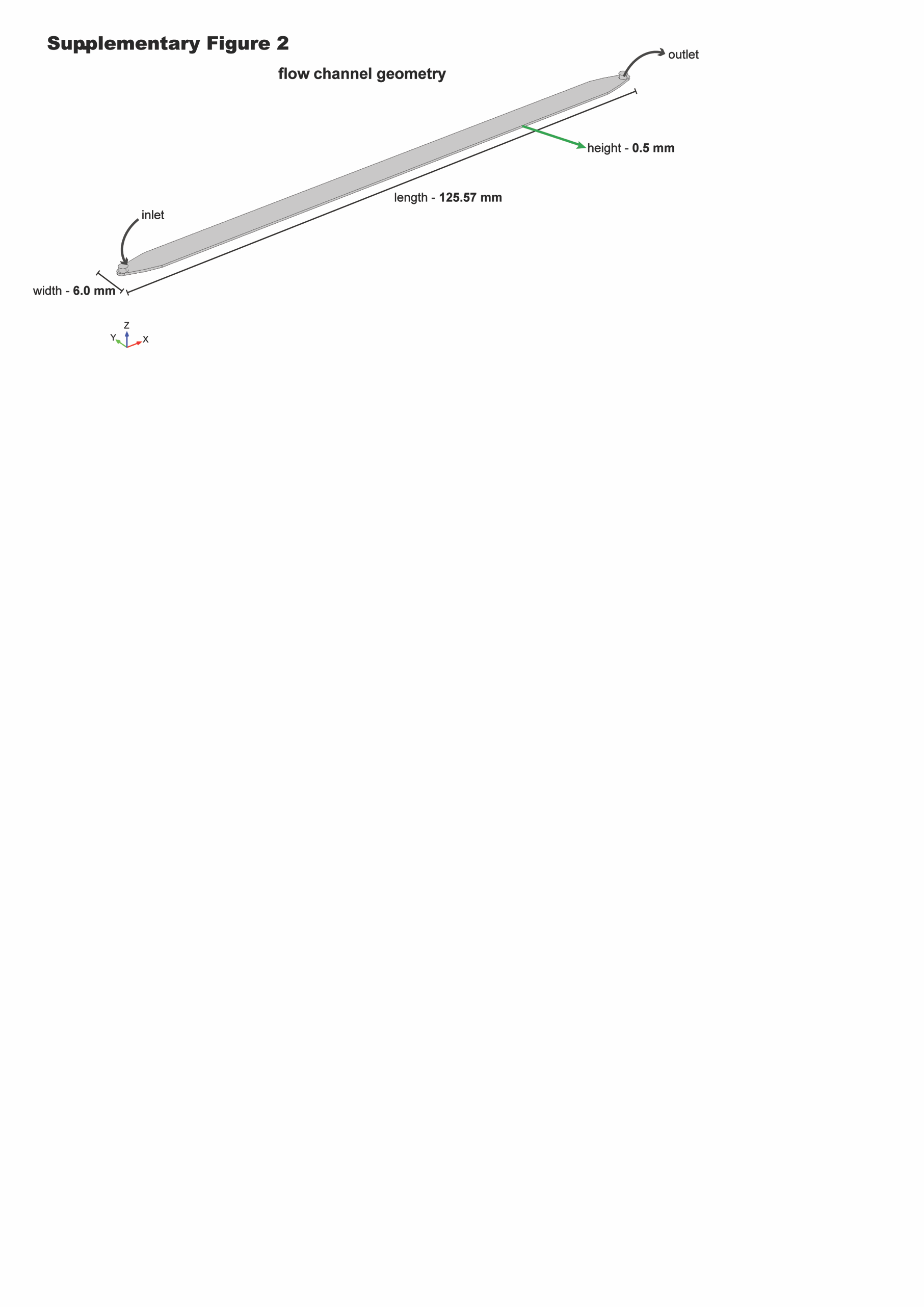
**

**Supplementary Figure 2. Flow channels geometries used on the COMSOL software to perform the simulations.**

Full channel size was simulated for this design. The inlet and outlet are identified by the black arrows and the flow channel by the green arrow. All the relevant measure are described in the figure. For the simulations, effect of tubing and connections is not considered.

**Supplementary Figure 3**


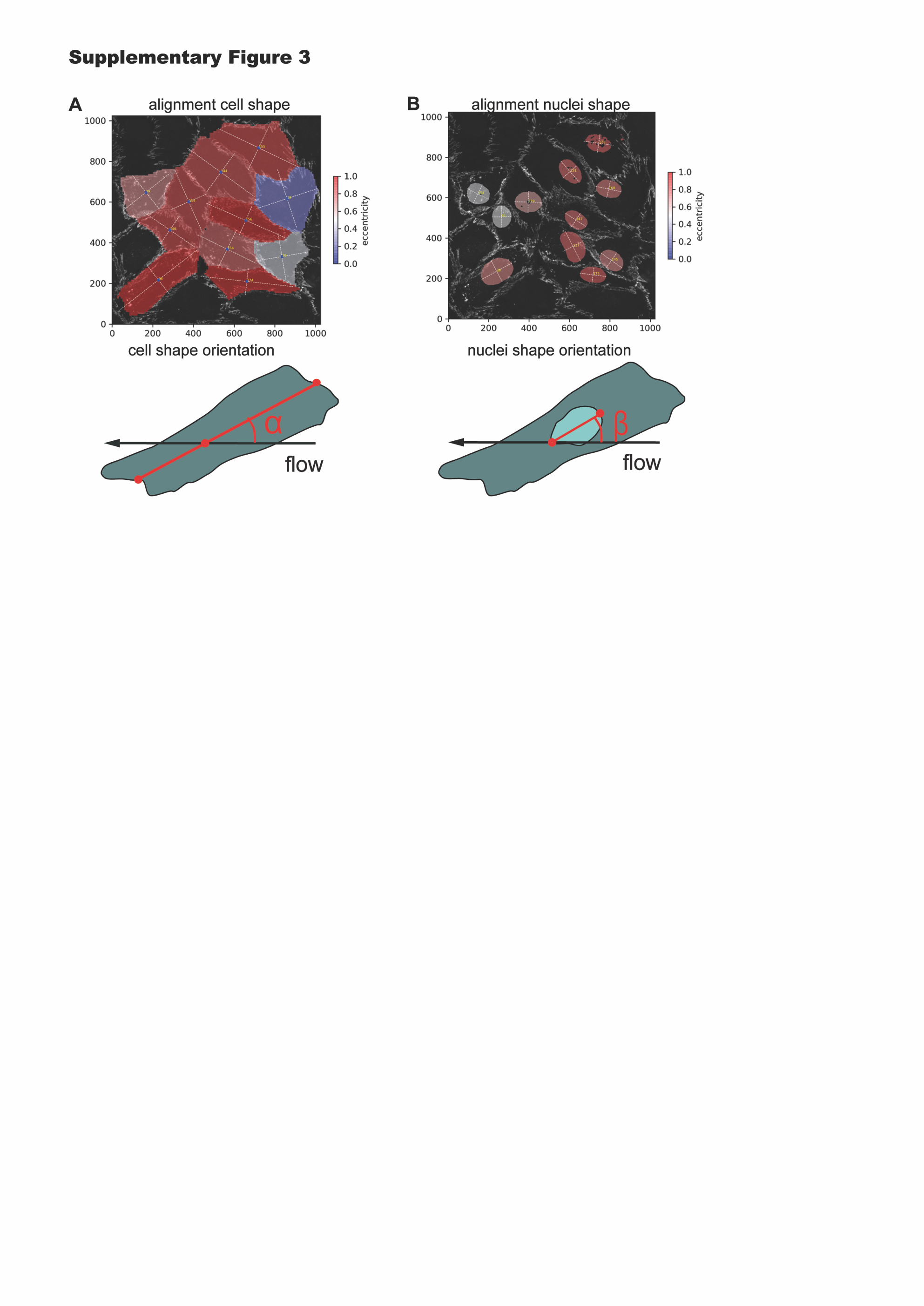


**Supplementary Figure 3. Assessment and analysis of HUVECs cell and nuclei shape and alignment.**

**(A)** Upper panel: Representative picture from Cellpose of cell shape segmentation using VE-cadherin as a proxy for cell outline. The eccentricity of the shape varies from 0 to 1 represented by the colors blue and red respectively. An eccentricity of 1 means that the longitudinal axis of the cell shape is longer than the horizontal axis representing an alignment of cell shape. Bottom panel: Schematic representation of the calculation method of endothelial cell shape orientation angles (α) in relation to the flow direction axis (x-axis). **(B)** Upper panel: Representative picture from Cellpose of nuclei shape segmentation using DAPI as a proxy for nuclei outline. The eccentricity of the nuclei shape varies from 0 to 1 represented by the colors blue and red respectively. An eccentricity of 1 means that the longitudinal axis of the nuclei shape is longer than the horizontal axis representing an alignment of nuclei shape. Bottom panel: Schematic representation of the calculation method of endothelial cell nuclei orientation angles (β) in relation to the flow direction axis (x-axis).

**Supplementary Figure 4**

**
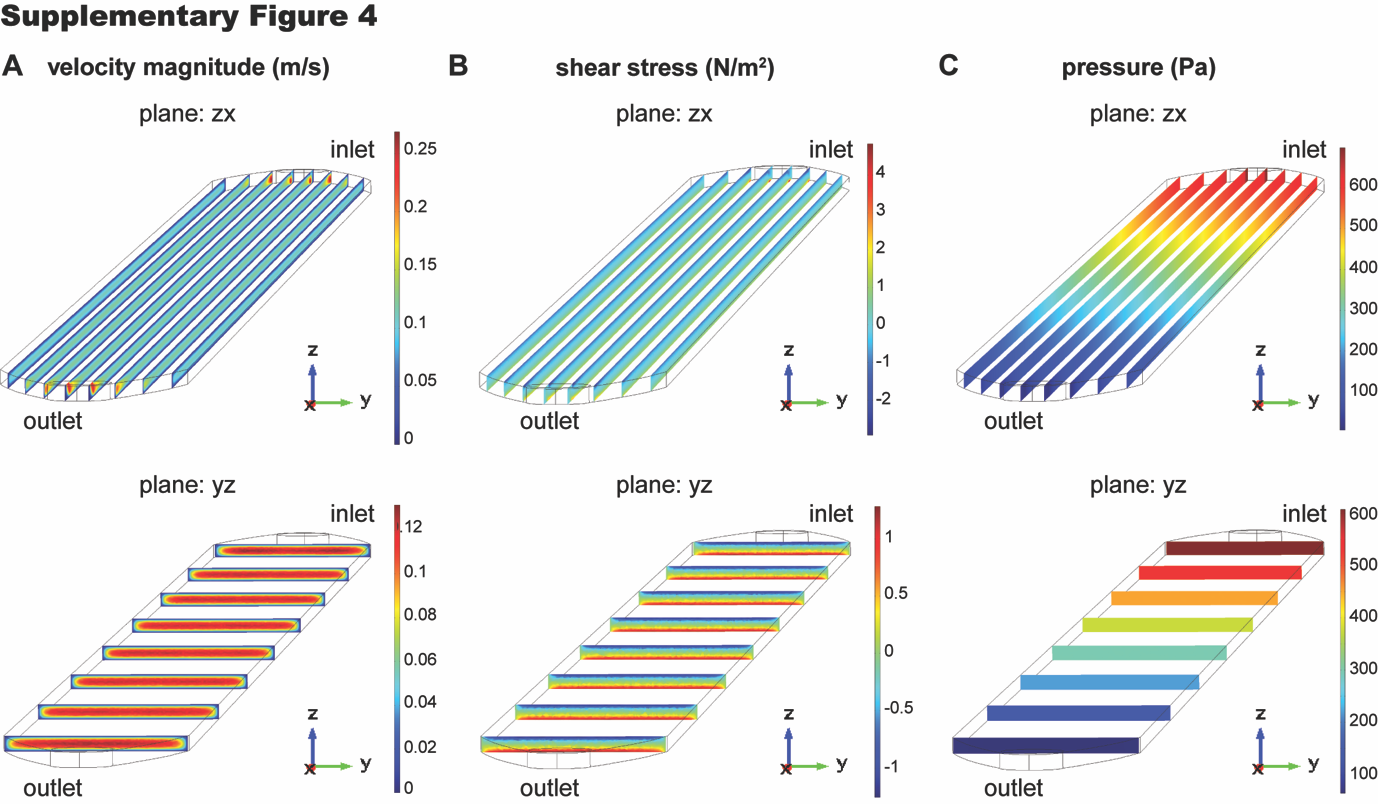
**

**Supplementary Figure 4. Computational Fluidic Dynamics in the 96-well fluidic channel for the xz, yz planes.**

**(A)** Simulation of the velocity magnitude (m/s) across the channel, in the zx plane (longitudinal section – Upper panel), and in the yz plane (transversal section – Bottom panel). **(B)** Simulation of the shear stress profile (N/m^2^) across the channel, in the zx plane (longitudinal section – Upper panel), and in the zx plane (transversal section– Bottom panel). **(C)** Simulation of the pressure profile (Pa) across the channel in the zx plane (longitudinal section – Upper panel), and in the yz plane (transversal section – Bottom panel). The simulations were performed for an initial velocity of 0.08m/s.
